# Structures of SARS-CoV-2 N7-methyltransferase with DOT1L and PRMT7 inhibitors provide a platform for new antivirals

**DOI:** 10.1371/journal.ppat.1011546

**Published:** 2023-07-31

**Authors:** Jithesh Kottur, Kris M. White, M. Luis Rodriguez, Olga Rechkoblit, Richard Quintana-Feliciano, Ahana Nayar, Adolfo García-Sastre, Aneel K. Aggarwal

**Affiliations:** 1 Department of Pharmacological Sciences, Icahn School of Medicine at Mount Sinai, New York, New York, United States of America; 2 Department of Microbiology, Icahn School of Medicine at Mount Sinai, New York, New York, United States of America; 3 Global Health and Emerging Pathogens Institute, Icahn School of Medicine at Mount Sinai, New York, New York, United States of America; 4 Department of Medicine, Division of Infectious Diseases, Icahn School of Medicine at Mount Sinai, New York, New York, United States of America; 5 The Tisch Institute, Icahn School of Medicine at Mount Sinai, New York, New York, United States of America; 6 Department of Pathology, Molecular and Cell-Based Medicine, Icahn School of Medicine at Mount Sinai, New York, New York, United States of America; Institut Pasteur, FRANCE

## Abstract

The RNA N7-methyltransferase (MTase) activity of SARS-CoV-2’s nsp14 protein is essential for viral replication and is a target for the development of new antivirals. Nsp14 uses S-adenosyl methionine (SAM) as the methyl donor to cap the 5’ end of the SARS-CoV-2 mRNA and generates S-adenosyl homocysteine (SAH) as the reaction byproduct. Due to the central role of histone MTases in cancer, many SAM/SAH analogs with properties of cell permeability have recently been developed for the inhibition of these MTases. We have succeeded in identifying two such compounds (SGC0946 and SGC8158) that display significant antiviral activity and bind to the SARS-CoV-2 nsp14 N7-MTase core. Unexpectedly, crystal structures of SGC0946 and SGC8158 with the SARS-CoV-2 nsp14 N7-MTase core identify them as bi-substrate inhibitors of the viral MTase, co-occupying both the SAM and RNA binding sites; positing novel features that can be derivatized for increased potency and selectivity for SARS-CoV-2 nsp14. Taken together, the high-resolution structures and the accompanying biophysical and viral replication data provide a new avenue for developing analogs of SGC0946 and SGC8158 as antivirals.

## Author summary

SARS-CoV-2 and its variants have led to millions of deaths globally. The RNA N7-methyltransferase activity of SARS-CoV-2’s nsp14 protein is essential for viral replication and represents a promising target for antiviral drug development. In this study, we identified two compounds (SGC0946 and SGC8158) with significant antiviral activity that bind to the SARS-CoV-2 nsp14 N7-MTase core. Co-crystal structures of these compounds with the N7-MTase core were determined, revealing features that can be modified for increased potency and selectivity. These findings provide an avenue for developing SGC0946/SGC8158 analogs as antivirals.

## Introduction

Emergence of the coronavirus SARS-CoV-2 has caused millions of deaths globally as well as lasting health problems in some who have survived the illness [1]. Along with vaccines [2], there is an urgent need to develop antivirals against this pernicious virus and its many variants. SARS-CoV-2 has a large (~ 30kb) positive-sense RNA genome that encodes 16 non-structural proteins as part of a polyprotein (nsp1 to nsp16) forming the replication-transcription complex (RTC)), as well as 4 structural proteins required for viral assembly and several accessory proteins that appear to modulate infection and pathogenesis [3]. The recent approval of oral antivirals such as molnupiravir and nirmatrelvir [4,5], targeting the RNA polymerase (nsp12) and main protease (M^Pro^/nsp5), respectively, has enabled new options for treating SARS-CoV-2 infection. However, a persistent concern with the wide use of these antivirals is the development of resistance [6,7]. There is thus an important need to develop additional antivirals targeting other enzymatic activities central to the survival and life cycle of the virus [3].

One such enzymatic activity is encoded by the SARS-CoV-2 nsp14 methyltransferase (MTase) domain, which, together with another MTase nsp16, caps the viral mRNAs for stability, efficient translation, and evasion of the host immune response [3]. Specifically, the nsp14 N7-MTase domain methylates the N7 atom of guanosine at the 5’end of mRNA to generate the ^N7Me^GpppA_2’OH_-RNA structure, which is then methylated at the 2’O atom of the initiating nucleotide by nsp16 to make the ^N7Me^GpppN_2’OMe_-RNA structure [3]. Both nsp14 and nsp16 use S-adenosyl methionine (SAM) as the methyl donor and generate S-adenosyl homocysteine (SAH) as the reaction byproduct.

The N7-MTase domain is located at the C-terminus of nsp14, whereas the N-terminus harbors an exoribonuclease domain (ExoN). Catalytic mutants of N7-MTase impair replication and abolish viral virulence, validating its significance as a SARS-CoV-2 therapeutic target [8,9]. To aid in the development of antivirals targeting the nsp14 N7-MTase activity, we recently reported high resolution crystal structures of the SARS-CoV-2 nsp14 N7-MTase core bound to SAM, SAH and Sinefungin (SFG; a general MTase inhibitor) [10]. The structures provide a basis for designing SAM/SAH mimetics that can compete with SAM to inhibit the methylation chemistry. However, one issue with “simple” SAM/SAH analogs as antivirals is that they tend to be highly hydrophilic and thus limited in their ability to cross the cell membrane [11]. But because of the central role of histone/protein methyltransferases in cancer, many SAM/SAH analogs have been developed in the cancer field with properties of cell permeability [11,12]. These analogs typically carry large hydrophobic groups and/or are modified as prodrugs to cross the cell membrane.

The SARS-CoV-2 nsp14 N7-MTase domain contains an atypical Rossmann fold and, amongst histone/protein methyltransferases, it is most similar in structure to the catalytic cores of DOT1L (a histone H3 lysine-79 MTase) and members of the PRMT family (protein arginine MTases) that contain a Rossmann fold. By focusing on SAM/SAH mimetics developed for DOT1L and members of the PRMT family, we have succeeded in identifying two compounds (SGC0946 and SGC8158) that 1) bind to SARS-CoV-2 nsp14, 2) co-crystallize with the N7-MTase core, and 3) display significant antiviral activity. We have solved structures of the N7-MTase core with both of these compounds which provide a framework for the structure guided design of new analogs with increased potency and selectivity for SARS-CoV-2 nsp14.

## Results

### Overall structures

For crystallization of the nsp14 N7-MTase core with SGC0946 and SGC8158, we employed the same protein-fusion strategy described previously for crystallization with SAM, SAH, and SFG [10]. That is, to assist crystallization, we fused a pH-sensitive mutant of the polymer-forming sterile alpha motif (SAM) [13,14] domain of human translocation ETS leukaemia (TEL) protein (TELSAM) (residues 47 to 124) to the MTase core, and then used the fusion protein (TEL-MTase) for co-crystallization with SGC0946 and SGC8158. As with SAM, SAH and SFG, this yielded co-crystals diffracting anisotropically to high resolution with synchrotron radiation, namely 1.57 Å for nsp14-MTase_SGC0946_ and 1.61 Å for nsp14-MTase_SGC8158._ The structures were determined by molecular replacement using the TEL-MTase from the nsp14-MTase_SAM_ structure (PDB id: 7TW7) as a search model (S1A and S1B Fig). The crystal data, data collection statistics, and refinement statistics for the two structures are summarized in Table 1.

**Table 1:** Data collection and refinement statistics.

	TELSAM-MTase_SGC0946_	TELSAM-MTase_SGC8158_
PDB ID	8FRJ	8FRK
**Data collection**		
Space group	P 65	P 65
Cell dimensions		
*a*, *b*, *c* (Å)	109.2 109.2 48.6	109.2 109.2 48.7
α, β, γ (°)	90.00 90.00 120.00	90.00 90.00 120.00
Resolution (Å)	54.6–1.57 (1.71–1.57)*	94.55–1.61 (1.86–1.61)*
*R*_sym_ or *R*_merge_	10.5 (111.6)	6.7 (93.5)
*I* / σ*I*	10.7 (1.6)	9.3 (2.0)
Completeness (ellipsoidal) (%)	94.3 (75.8)	94.3 (68.6)
Redundancy	10.2 (7.1)	7.0 (7.0)
CC(1/2)	0.99 (0.62)	1.0 (0.68)
**Refinement**		
Resolution (Å)	36.31–1.57	47.3–1.61
No. reflections	31716	23242
*R*_work_ / *R*_free_	17.0/20.4	21.7/25.1
No. atoms		
Protein	2087	2070
Ligands	40	40
Water	386	246
*B*-factors		
Protein	25.41	33.13
Ligand/ion	33.75	49.00
Water	40.34	43.37
R.m.s. deviations		
Bond lengths (Å)	0.01	0.006
Bond angles (°)	0.93	0.78
Ramachandran Plot		
Favored (%)	97.67	97.6
Allowed (%)	2.33	2.40
Outliers (%)	0.00	0.00

*Values in parentheses are for highest-resolution shell.

**Fig 1:**
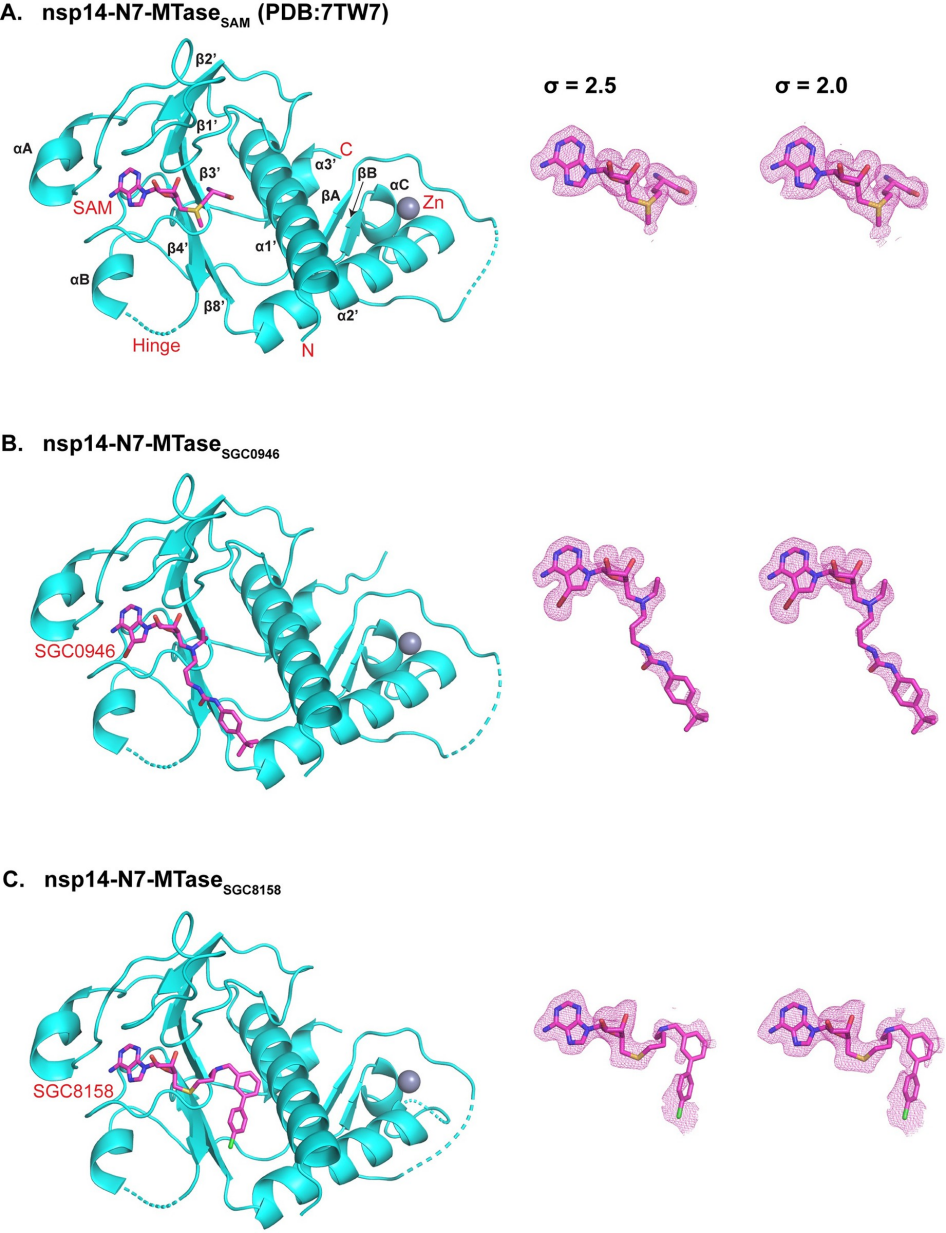
Structures of nsp14-N7-MTase bound to ligands. **(A)** Structure of nsp14-N7-MTase bound to SAM (PDB id: 7TW7) (left). The secondary structure elements are marked. The polder maps (right) for the SAM are shown in pink and contoured at 2.5σ and 2.0σ. **(B)** Structure of nsp14-N7-MTase bound to SGC0946 (left). The polder maps (right) for the SGC0946 are shown in pink and contoured at 2.5σ and 2.0σ. **(C)** Structure of nsp14-N7-MTase bound to SGC8158 (left). The polder maps (right) for the SGC8158 are shown in pink and contoured at 2.5σ and 2.0σ. The residues not modeled in the structure are shown by dashed lines. A zinc ion (Zn) is shown as a sphere and colored grey.

**Fig 2:**
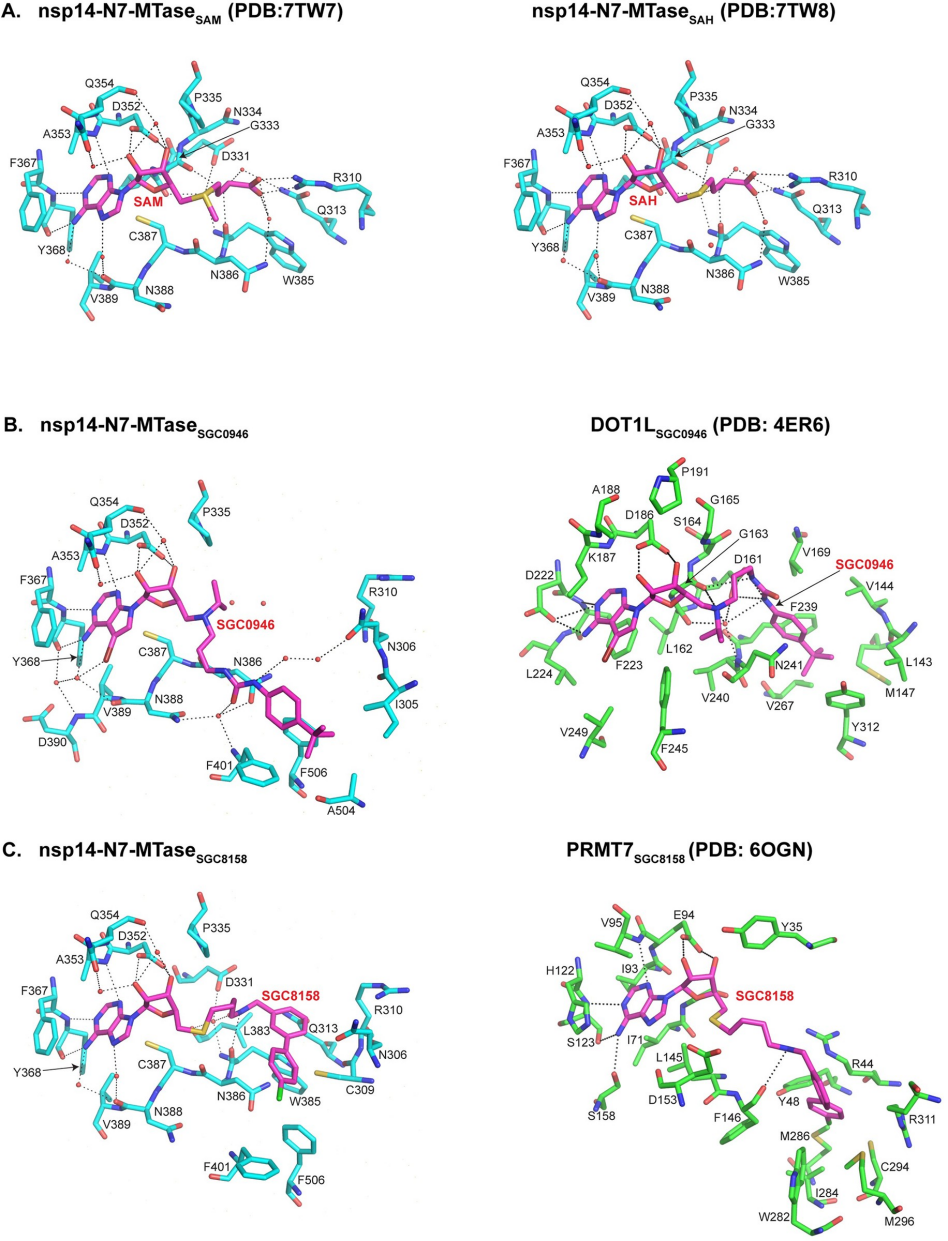
Detailed representation of structures of SARS-CoV-2 nsp14-N7-MTase bound to ligands. **(A)** Detailed view of interactions of nsp14 MTase domain bound to SAM (left) and SAH (right). Hydrogen bonds between the MTase domain and SAM/SAH are depicted as dashed lines and the water molecules are shown as red spheres. **(B)** Detailed view of interactions of nsp14 MTase domain bound to SGC0946 (left, cyan). The interactions of DOT1L bound to SGC0946 are shown on the right (green). **(C)** Detailed view of interactions of nsp14 MTase domain bound to SGC8158 (left, cyan). The interactions of PRMT7 bound to SGC8158 are shown on the right (green).

Overall, the MTase core consists of an atypical Rossmann fold with five central β-strands (β1’, β2’, β3’, β4’ and β8’) instead of the usual seven (β1-β7). Helices α1’, α2’, α3’ and αC, β-strands βA and βB, and a Zn^2+^ coordinated substructure are located on one side of this central β-sheet, and two short helices αA and αB are on the other. SGC0946 and SGC8158 are located at the C-terminal ends of strands β1’, β2’, β3’, and are supported by loops between β1’ and β2’, β2’ and αA, and β3’ and β4’ (Fig 1A–1C). The nsp14-MTase_SGC0946_ and nsp14-MTase_SGC8158_ structures are very similar to those reported previously with SAM, SAH and SFG, with r.m.s deviations of ~0.08 to 0.16 Å (for 164 Cα atoms), marking a surprisingly minimal change in the protein conformation in accommodating the bulky hydrophobic groups of SGC0946 and SGC8158 (Figs 1 and S1C). A notable exception is the Arg310 side chain, which orients to make hydrogen bonds with the methionine portion of SAM, SAH or SFG, but in the nsp14-MTase_SGC0946_ and nsp14-MTase_SGC8158_ structures it orients away from the cofactor binding site (Fig 2A–2C).

Interestingly, in the full-length coronavirus nsp14 structures [15,16], a characteristic of the MTase fold is a “hinge” region composed of a three stranded β-sheet (residues 402–433) and an interdomain loop (residues 288–299) that precedes the MTase core. This β-sheet is disordered in our structures, suggesting that its interactions with the ExoN domain are required for its folding and stability.

### Mode of SGC0946 binding

The bromo-deazaadenosine moiety of SGC0946 is well-defined in the electron density map and overlays with the adenosine portion of SAM in the nsp14-MTase_SAM_ structure [10] (Fig 1B). In a similar manner to the adenosine of SAM (Fig 1A), the bromo-deazaadenine ring is accommodated in a cavity formed by the Ala353, Phe367, Tyr368, Cys387 and Val389 side chains, with its N1 and N6 atoms making hydrogen bonds with the backbone amide and carboxyl groups of Tyr368, respectively, and the ribose sugar making direct hydrogen bonds with the Asp352 side chain, as well as water mediated interactions with both the Gln354 side chain and main chain (Fig 2B). The bulky t-butylphenyl portion of SGC0946 is less defined in the electron density map, indicative of flexibility when binding to the SAM site (Fig 1B). The best interpretation of the electron density places t-butylphenyl in a small hydrophobic cavity delineated by residues Ile305, Phe401, Ala504 and Phe506 on the nsp14 MTase core surface (Fig 2B). Intriguingly, this cavity is the site of RNA binding; for example, in the structure of SARS-CoV nsp14/nsp10 with SAH and GpppA, the guanine of GpppA is accommodated in this cavity (S2A Fig). This strongly alludes to SGC0946 as a bi-substrate inhibitor of SARS-CoV-2 nsp14: co-occupying both the SAM and the GpppA cap binding sites and as a novel scaffold for the design of potent antivirals targeting both SAM and RNA binding.

Interestingly, the extended urea and isopropyl ammonium between the bromo-deazaadenosine and the t-butylphenyl portions of SGC0946 is largely devoid of direct interactions with the nsp14 MTase core. The most significant interactions are with water molecules, wherein the urea linkage, for example, makes water mediated interaction with Asn306, Asn386, Asn388 and Phe401 (Fig 2B). However, there may be some hydrophobic contacts from the hinge region, which is disordered in our structure. That is, when we align nsp14-MTase_SGC0946_ with the full-length nsp14 structure [16] (PDB id: 7R2V), residues Trp292, Leu409 and Phe426 from the hinge are in positions to putatively interact the isopropyl ammonium or the t-butylphenyl group of SGC0946, but Tyr420 would need to rearrange on SGC0946 binding (S2B Fig). Overall, the urea/isopropyl ammonium linkage offers opportunities for derivatization for additional interactions with the SARS-CoV-2 N7-MTase.

Compared to the DOT1L_SGC0946_ structure [17], the t-butylphenyl group and the urea-isopropyl linkage in our structure occupy different positions relative to the deazaadenosine. These moieties are also involved in much more extensive interactions with DOT1L, whereby the t-butylphenyl group fits in an extended hydrophobic cavity delineated by Leu143, Val144, M147, V169, Phe239, Asn241, Val267 and Tyr312, and the urea linkage partakes in a network of direct and water mediated interactions with Asp161, Leu162, Gly163 and Asn241 (Fig 2B). SGC0946 has also been crystallized with the DNA adenine MTase CamA that also contains a Rossmann fold [18]. The linker and the t-butylphenyl group adopt yet another configuration when bound to CamA. Strikingly, the t-butylphenyl group points in three very different directions in the nsp14, DOT1L and CamA structures, indicating the high flexibility of SGC0946 in binding to functionally different MTases (S2C Fig).

### Mode of SGC8158 binding

The adenosine portion of SGC8158 is well-defined in the electron density map and it makes almost identical interactions as the adenosine moiety of SAM, whereby the adenine ring fits in a cavity delineated by the Ala353, Phe367, Tyr368, Cys387 and Val389 side chains, with its N1 and N atoms making hydrogen bonds with the backbone amide and carboxyl groups of Tyr368, respectively (Figs 1C and 2C). Also, the ribose sugar of the adenosine makes direct hydrogen bonds with the Asp352 side chain and water mediated interactions with both the Gln354 side chain and main chain. SGC8158 diverges from SAM beyond the adenosine core and the terminal portion containing the chlorobiphenyl moiety is not well-defined in electron density, indicating flexibility. The best interpretation of the electron density (and subsequent refinement) shows the chlorobiphenyl moiety making van der Waals and hydrophobic interactions with Trp385 and Phe506 and overlapping with the putative RNA binding site (S2D Fig). The moiety could be further stabilized in the context of full-length nsp14; for example, when we align the structure with full-length nsp14 [16] (PDB id: 7R2V), residues Trp292, Tyr420 and Phe426 from the hinge region can putatively interact with SGC8158 (S2E Fig). Analogous to SGC0946, SGC8158 emerges as a bi-substrate inhibitor of SARS-CoV-2 nsp14, co-occupying both the SAM and GpppA cap binding sites.

Compared to the MTase core in the nsp14-MTase_SAM_ structure [10], the most significant conformational change is in the positions of Arg310 and Phe401 side chains. Arg310 swings away from binding site to avoid steric overlaps with the biphenyl moiety, and the Phe401 ring shifts by ~0.8Å towards the chlorobiphenyl moiety to make van der Waals contacts with the chlorine atom (Fig 2A and 2C).

Compared to the PRMT7_SGC8158_ structure [19], the conformation of the chlorobiphenyl moiety and the preceding methylamine linker is strikingly different. In PRMT7, the chlorobiphenyl moiety interacts more extensively with the enzyme and occupies a cavity formed by Arg44, Tyr48, Phe146, Trp282, Ile284, Met286 and Cys294 (Fig 2C). SGC8158 has also been crystallized with the DNA adenine MTase CamA [18], in which the methylamine linker is almost fully extended and chlorobiphenyl moiety occupies a position different again from that observed with nsp14 and PRMT7 (S2F Fig). Analogous to SGC0946, SGC8158 emerges as a highly malleable compound that can readily mold to the cofactor binding sites of functionally different MTases.

### SGC0946 and SGC8158 bind the nsp14/nsp10 complex with micromolar affinities

**Fig 3:**
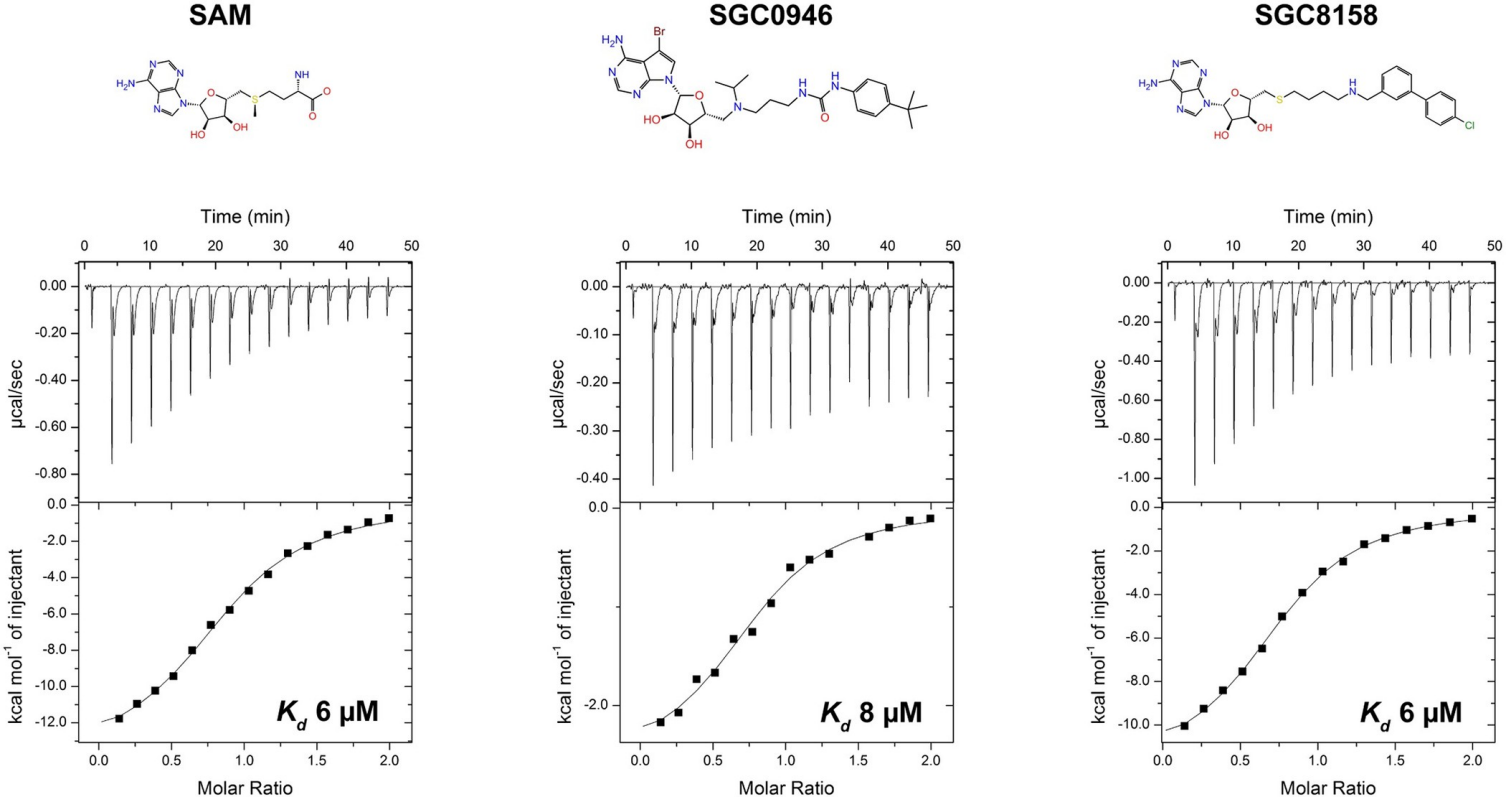
ITC binding analysis of nsp14/nsp10 complex with SAM, SGC0946 and SGC8158. ITC titration data for the nsp14/nsp10 complex for SAM (left), SGC0946 (center), and SGC8158 (right). The resultant binding isotherms were used to calculate the equilibrium dissociation constants (*K*_D_). Chemical structure for each ligand is also shown.

We used isothermal titration calorimetry (ITC) to determine and compare the thermodynamic parameters of SGC0946 and SGC8158 binding to the SARS-CoV-2 nsp14/nsp10 complex. The complex was purified from *E*.*coli* cells after transformation with a pRSF-duet-1 plasmid bearing C-terminal 6xHis-tagged full-length nsp14 and nsp10. SAM binds nsp14/nsp10 with a similar equilibrium dissociation constant *K*_*d*_ value of ~6 μM as previously reported [10]. Interestingly, SGC0946 and SGC8158 bind to nsp14/nsp10 with similar affinities as SAM, with *K*_*d*_ values of ~8 μM and ~6 μM, respectively (Fig 3). Importantly, from the ITC data, the bulky hydrophobic groups of SGC0946 and SGC8158 do not impede their binding to nsp14 and both compounds are good starting point for the development of more effective and targeted inhibitors.

### SGC0946 and SGC8158 inhibit the binding of RNA cap analog

**Fig 4:**
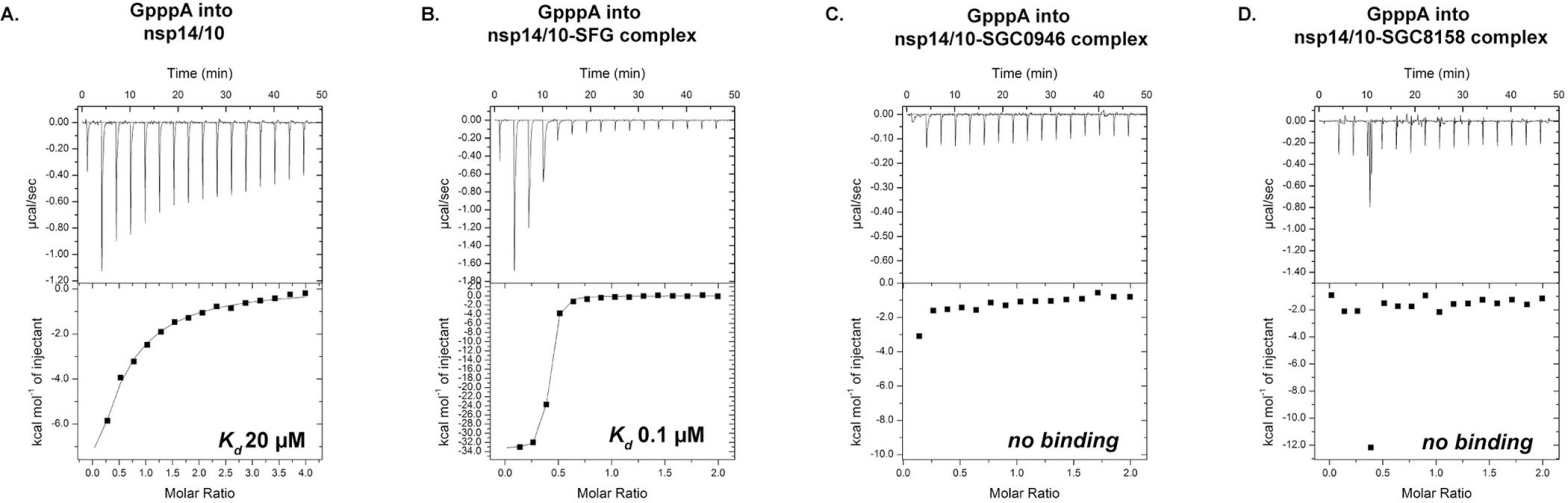
ITC binding analysis of nsp14/nsp10 complex with GpppA RNA cap analog in the absence and presence of ligands. ITC titrations of GpppA RNA cap analog into the nsp14/nsp10 complex **(A)**, nsp14/nsp10-SFG complex **(B)**, nsp14/nsp10-SGC0946 complex **(C)** and nsp14/nsp10-SGC8158 complex **(D)** are displayed. In the presence of inhibitors SGC0946 and SGC8158, binding of the RNA cap analog is inhibited.

An interesting feature of SGC0946 and SGC8158 is their co-occupancy of both the SAM and the putative RNA binding sites in nsp14. To test this further, we conducted ITC binding analysis of GpppA RNA cap analog to the nsp14/10 complex in the absence and presence of both inhibitors. The ITC analysis affirms the dual occupancy observed in the crystal structures. In the absence of inhibitors, the cap analog binds to nsp14/10 with an affinity of 20μM, but there is no binding in the presence of either SGC0946 or SGC8158 (Fig 4). (Intriguingly, RNA cap binds 200-fold better to the nsp14/10-Sinefungin complex than nsp14/10 alone). Taken together, SGC0946 and SGC8158 offer the prospect of developing inhibitors of SARS-CoV-2 nsp14 that compete with both SAM and RNA binding.

### Inhibition SARS-Cov-2 replication in HeLa-ACE-2 cells

To see whether the binding of SGC0946 and SGC8158 to nsp14 inhibits SARS-CoV-2 replication in vivo, we used HeLa-ACE2 cells, a cell line permissive to SARS-CoV-2 infection. Importantly, for these cellular studies we employed a prodrug version of SGC8158, known as SGC3027. This is because SGC8158 only enters cells efficiently as the prodrug SGC3027. SGC3027 was designed by Szewczyk et al [19] to increase the cell permeability of SGC8158 by employing the Trimethyl Lock strategy, wherein the compound was derivatized with a quinonebutanoic acid to mask the secondary amine group and to increase lipophilicity. Once inside cells, SGC3027 undergoes reduction and lactonization to release the active SGC8158 form [19]. Thus, all of our structural and binding studies (described above) have been performed with active SGC8158 form, but for cell-based viral assays we employed the prodrug SGC3027.

**Fig 5:**
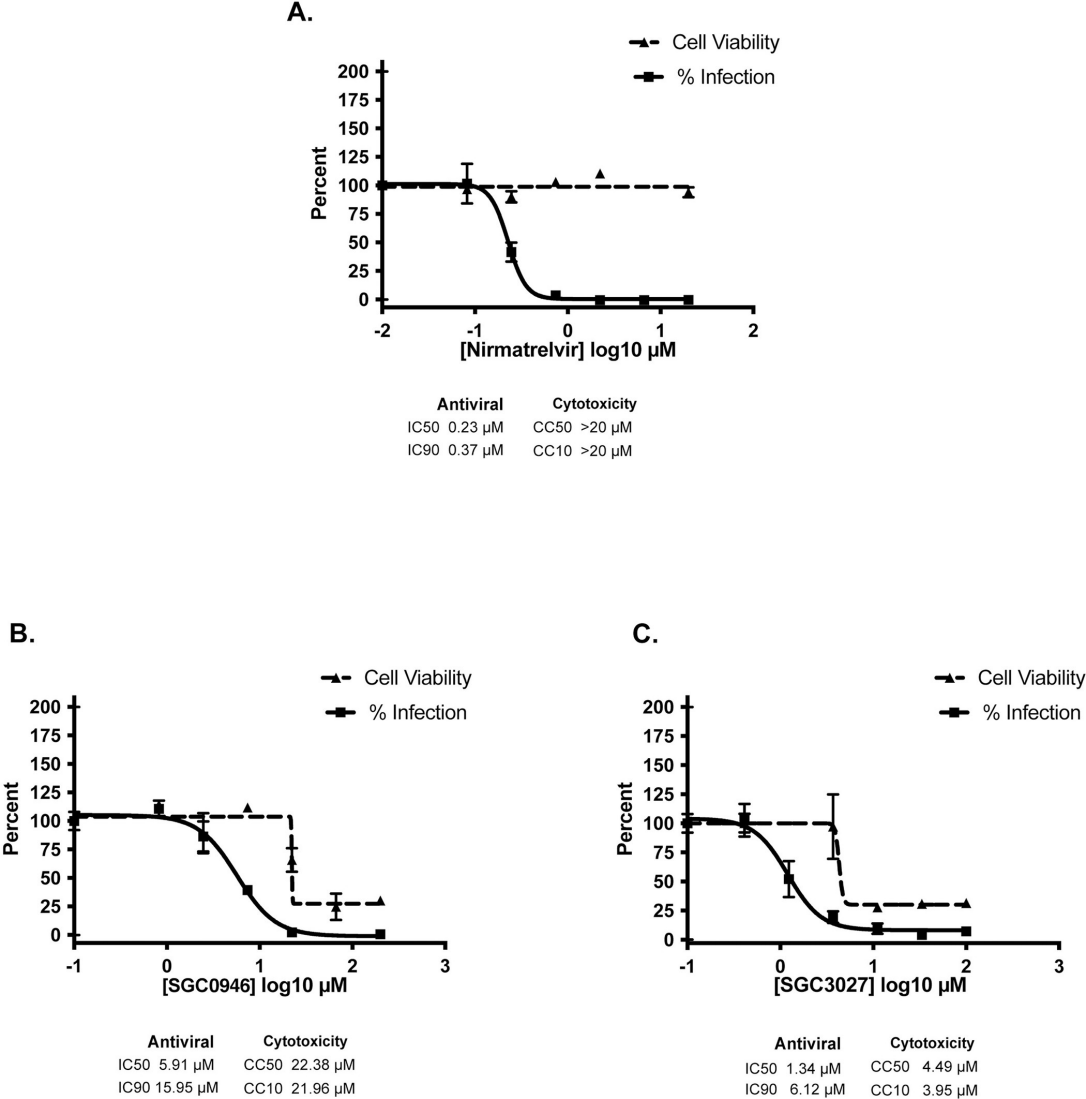
SGC0946 and SGC3027 inhibits SARS-CoV-2 in HeLa-ACE2 cells. Dose-response analysis of HeLa-ACE2 cells with Nirmatrelvir **(A)**, SGC0946 **(B)** and SGC3027, prodrug version of SGC8158 **(C)**.

Briefly, the viral assays with SGC0946 and SGC3027 were performed in a BSL3 facility at Mount Sinai. All assays were performed in biologically independent triplicate and included Nirmatrelvir, Pfizer’s emergency-approved COVID-19 antiviral, and DMSO controls. Fig 5 shows the results for SGC0946 and SGC3027. Importantly, both compounds inhibit SARS-CoV-2 replication at concentrations significantly lower than that cause cytotoxicity, and their IC50 values are in the range observed for binding to nsp14 observed by ITC. Indeed, the IC50 values for SGC3027 and SGC0946 are only ~ 6 to 26-fold higher than that for the FDA approved antiviral nirmatrelvir (IC50 of ~0.23μM, Fig 5). The major difference between SGC0946 and SGC3027 and nirmatrelvir is their toxicity at higher concentrations (Fig 5). However, this toxicity is not unexpected as SGC0946 and SGC3027 were selected and optimized for DOT1L and PRMT7, respectively, which play essential roles in a wide variety of normal cellular processes [20,21]. This toxicity will improve as we optimize SGC0946 and SGC3027 for binding to SARS-CoV-2 nsp14 rather than DOT1L or PRMT7.

## Discussion

SARS-CoV-2 and its variants have led to millions of deaths globally [1]. Effective vaccines have been developed to contain the virus [2], but there is an urgent need to develop antivirals that can be disseminated quickly and widely and used for the treatment of patients already infected. The FDA has recently approved the use of oral antivirals molnupiravir and nirmatrelvir [4,5], but as with most antivirals, there is persistent concern of the virus evolving resistance to these drugs upon wide use [6,7]. Accordingly, there is an important need to develop additional antivirals targeting other enzymatic activities central to the survival and life cycle of the virus [3]. One such enzymatic activity is encoded by the SARS-CoV-2 nsp14 N7-MTase domain, which uses SAM as a cofactor to methylate the 5’-end of viral mRNA, as part of a group of viral enzymes that “cap” the mRNA for stability, efficient translation, and evasion of the host immune response.

Compounds that inhibit the SARS-CoV-2 nsp14 MTase activity by competing with SAM present an attractive strategy for the development of antivirals. Although, “simple” SAM-mimetics with nanomolar affinities for the SARS-CoV-2 nsp14 MTase have been identified [22,23], they remain to be tested in a cellular context. One issue with simple SAM-mimetics is their hydrophilicity that hampers their ability to cross cell membranes. Fortunately, many SAM analogs have been developed in the cancer field for inhibiting histone methyltransferases and which are well tested in cell lines [11,12]. We conducted a screen of commercially available SAM-based inhibitors of PRMTs and DOT1L using ITC and viral replication assays, and identified SGC0946 and SGC8158 as the most potent compounds. Here, we report high-resolution crystal structures of the SARS-CoV-2 nsp14 MTase core with both SGC0946 and SGC8158. We also show that SGC0946 and SGC8158 are bi-substrate inhibitors of nsp14 and possess significant antiviral activity in HeLa-ACE2 cells.

SGC0946 and SGC8158 are potent inhibitors of DOT1L (a lysine MTase) and PRMT7 (an arginine MTase), respectively; both in vitro and in vivo [17,19]. The DOT1L_SGC0946_ and PRMT7_SGC8158_ crystal structures provide bases for their potency—revealing extensive non-polar and polar interactions [17,19]. The nsp14-MTase_SGC0946_ and nsp14-MTase_SGC8158_ crystal structures reveal the deazadenosine/adenosine “head” portions of SGC0946 and SGC8158 binding to the nsp14 MTase core similarly to the protein MTases, but their hydrophobic “tail” portions assume very different configurations than those observed with the protein MTases. This alludes to the extraordinary malleability of SGC0946 and SGC8158 in binding to functionally different MTases.

SGC0946 and SGC8158 bind to DOT1L and PRMT7, respectively, with *K*_*D*_*’*s in the nanomolar range, as compared to the low micromolar range determined here for nsp14 [17,19]. This is not unexpected as SGC0946/SGC8158 were selected and optimized for binding to DOT1L/PRMT7 and their tail portions in particular make many more interactions with these selected enzymes [17,19]. The paucity of interactions with nsp14 does, however, present opportunities to derivatize SGC0946 and SGC8158 for additional interactions with nsp14 for inhibition of both SAM and RNA binding. For example, the aliphatic portion of SGC0946 bridging the isopropyl ammonium and urea linkage lies close to the main chain carbonyl of Asn386 and could be potentially modified to make hydrogen bonds with it. Similarly, the chlorobiphenyl moiety of SGC8158 lies in close proximity to a number of polar or charged residues, including Asn306, Arg310, Glu313, and Asn386, and could be derivatized for hydrogen bonding with these residues. Another modification would be the addition of a large aromatic group such as a quinoline to the nucleobase of SGC0946 and SGC8158/SGC3027. (SGC3027 is the prodrug version of SGC8158 for cell permeability–see above). This follows from a finding by Otava et al. [23] that the addition of a quinoline via an ethynediyl bridge to the C7 position of a deazadenine derivative of SAH results in a compound (TO507) with nanomolar affinity (~17.9 nM) for SARS-CoV-2 nsp14 MTase. We anticipate that SGC0946 and SGC8158/SGC3027 derivatized as such will bind with higher affinity and selectivity to SARS-CoV-2 nsp14. SGC8158/SGC3027 also present the intriguing prospect of designing a covalent inhibitor of nsp14. Notably, chlorobiphenyl moiety of SGC8158 lies close to Cys309 of nsp14, allowing for a suitable “warhead” in the moiety to make a covalent bond with the cysteine. Covalent inhibitors have proven highly efficacious in the inhibition of kinases, oncogenic KRAS G12C, as well as MTases [24].

Importantly, SGC3027 and SGC0946 inhibit SARS-CoV-2 replication at concentrations significantly lower than that cause cytotoxicity, with IC50 values only ~ 6 to 26-fold higher than that for the FDA approved antiviral nirmatrelvir (IC50 of ~0.23μM). SGC0946 and SGC3027 differ from nirmatrelvir primarily in their toxicity at higher concentrations, but this is not unexpected as SGC0946 and SGC8158/SGC3027 were selected and optimized for DOT1L and PRMT7, respectively, which play essential roles in a wide variety of normal cellular processes [20,21]. This toxicity will improve as we optimize SGC0946 and SGC8158/SGC3027 for selective binding to SARS-CoV-2 nsp14 rather than DOT1L or PRMT7. For example, the addition of quinoline to the nucleobase of SGC8158/SGC3027 provides a basis for higher affinity binding to SARS-CoV-2 nsp14 but it severely clashes with the PRMT7 active site. Similarly, modifications to the aliphatic portion of SGC0946 and the chlorobiphenyl moiety of SGC8158/SGC3027 to make additional hydrogen bonds with nsp14 residues are incompatible with the DOT1L and PRMT7 active sites. Also, there is no equivalent of nsp14 Cys309 in PRMT7 to permit a covalent attachment to a designed warhead on SGC8158/SGC3027. There has been an effort to develop simple selective SAM analogs against Dengue and other flavivirus MTases [25]. The advantage that SGC8158/SGC3027 and SGC0946 offer as starting points for inhibitors of viral MTases (besides their membrane permeability) is their extended backbones that span the entire width of the SARS-CoV-2 nsp14 active site—blocking both SAM and RNA binding—and presenting many more opportunities to build selectivity for SARS-CoV-2 nsp14 over host methyltransferases.

In summary, we identify SGC0946 and SGC8158/SGC3027 as novel scaffolds for developing antivirals against SARS-CoV-2. Both compounds bind SARS-CoV-2 nsp14 with affinities comparable to SAM and display significant antiviral activity. The structures presented provide a platform for modifying SGC0946 and SGC8158/SGC3027 for increased potency and selectivity for inhibition of SARS-CoV-2 nsp14.

## Methods

### Protein expression and purification

#### Full-length nsp14/nsp10 complex

The protein was purified as previously [10]. Briefly, transformed *Escherichia coli* BL21Gold (DE3) cells with single pRSF-duet-1 plasmid bearing C-terminal 6xHis-tagged full-length nsp14 and nsp10 were grown in LB media. The protein expression was induced by adding 0.5mM IPTG and incubated at 15°C for 18hours. The cells were harvested and resuspended in binding buffer (25mM Tris pH 7.5, 250mM NaCl, 10% glycerol, 0.01% IGEPAL, 25mM imidazole, 10μM ZnCl_2_ and 10mM 2-mercaptoethanol). The cells were lysed by sonication and the supernatant was loaded onto a HisTrap HP affinity column (GE Healthcare). The column was washed with binding buffer and then the complex was eluted using buffer with 500mM imidazole. The eluted protein was further subjected to size exclusion chromatography with a buffer containing 100mM KH_2_PO_4_/K_2_HPO_4_ buffer pH 8.0, 100mM KCl, 0.01% IGEPAL, 5mM 2-mercaptoethanol and 10% Glycerol.

#### TEL-MTase

The protein was purified as described previously [10]. Briefly, nsp14 MTase domain (AA300-527) was fused to TELSAM (AA47-124) with a PAA linker. The pRSF-Duet-1-smt3 plasmid containing N-terminal 6xHis-SUMO-TEL-PAA-MTase was transformed into *Escherichia coli* BL21Gold (DE3) cells. The cells were grown in LB media and at OD_600_~0.8, 0.5mM IPTG was added and reduced the temperature to 15°C. The cells were harvested after 18 hours of incubation and resuspended in binding buffer (25mM Tris pH 7.5, 500mM NaCl, 10% glycerol, 0.05% IGEPAL, 30mM imidazole, 10μM ZnCl_2_ and 10mM 2-mercaptoethanol). The cells were lysed and subjected to affinity, ion-exchange, and size exclusion chromatography. The final purified protein was stored in 25mM Tris pH 8.3, 200mM KCl and 2mM TECP.

### Isothermal titration calorimetry (ITC)

The ITC experiments were performed with a Microcal ITC_200_ instrument at 25°C. SAM/SGC0946/SGC8158/GpppA cap analog (NEB # S1406S) were loaded in the syringe (500μM) and titrated into 50μM of nsp14/nsp10 complex in the cell. The titrations were performed with the standard 10μcals/s reference power and at 600rpm. The titrations with SGC0946 and SGC8158 do not reach a full plateau due to the insolubility of the compounds at higher concentrations. In the case of RNA cap analog binding measurements in the presence of ligand, the protein was incubated with 200uM ligands (SFG/SGC0946/SGC8158) then 500μM GpppA cap analog was titrated into the nsp14/10-ligand complex. Origin 7.0 software was used to fit the data to a single binding site model. All the experiments were repeated twice and the average values reported.

### Viral assays

For SARS-CoV-2 antiviral assays HeLa-ACE2 cells were seeded in 96-well plates at 2,000 cells/well for 24 hours at 37°C, 5% CO_2_. Two hours prior to infection, medium (DMEM 10% FBS) was replaced with 100ul infection medium (DMEM 2% FBS) containing the compound of interest at concentrations 50% greater than those indicated, including a DMSO control. Plates were then transferred into the BSL3 facility, where they were then infected with 1000 PFU (HeLa-ACE2 MOI = 0.25) of the Wuhan-like SARS-CoV-2/WA1 variant in 50ul infection medium, bringing the final compound concentration to those indicated. Plates were then incubated at 37°C for 48 hours before being fixed with 10% formaldehyde for 24 hours. Plates were then removed from the BSL3 facility and immunostained for the viral NP protein using an inhouse mAb 1C7, provided by Dr. Thomas Moran (Thomas.Moran@mssm.edu) with a DAPI counterstain. Infected cells (488 nm) and total cells (DAPI) were quantified using the Celigo (Nexcelcom) imagine cytometer. Infectivity was quantified as ((Infected cells/Total cells) -Background)*100, with the DMSO control set to 100% infection for analysis. Concurrent cytotoxicity assays using the MTT assay (Roche) were performed in uninfected HeLa-ACE2 cells with the same compound dilutions. All assays were performed in biologically independent triplicate and included Nirmatrelvir, Pfizer’s emergency-approved COVID-19 antiviral, and DMSO controls. Data was fit using nonlinear regression and full 6-point SARS-CoV-2 antiviral curves were generated for all compounds using Prism GraphPad version 8.0.2 (San Diego, CA).

### Crystallization and structure determination

The binary complexes were reconstituted by mixing 15mg/ml TEL-MTase fusion protein with 5-fold molar excess of the ligand (SGC0946 and SGC8158). The crystals were grown in 6–10% Reagent Alcohol, 0.2–0.4 M Lithium Sulfate and 0.1M Sodium Citrate pH 5.5–6.3 in 2 days at 20°C. The crystals were cryoprotected with reservoir solutions containing 30% glycerol. X-ray diffraction data were collected at the NSLS-II 17-ID-1 and 17-ID-2 beamlines at the Brookhaven National Laboratory (BNL) under cryogenic conditions.

The diffraction data were processed using DIALS and AIMLESS in the CCP4 suite [26,27]. The anisotropic correction was performed using the STARANISO server (https://staraniso.globalphasing.org/cgi-bin/staraniso.cgi) with a surface threshold of I/σ(I) ≥ 1.2. The structures were solved by molecular replacement with Phaser-MR [28] using 7TW7 as the search model. Subsequent iterative manual building and refinement were performed with Coot and Phenix refine, respectively [29,30]. Ligand restraint file for SGC0946 and SGC8158 were generated using eLBOW [31] from the PHENIX suite. All molecular graphic figures were prepared using PyMOL (Schrödinger LLC).

## Supporting information

S1 Fig Overall structures of nsp14-N7-MTase-compound complexes. **(A)** Overall structure of SARS-CoV-2 nsp14-N7-MTase_SGC0946_ complex fused with TELSAM. The nsp14 N7-MTase domain and TELSAM are colored in cyan and yellow, respectively. **(B)** Overall structure of SARS-CoV-2 nsp14-N7-MTase_SGC8158_ complex fused with TELSAM. **(C)** Cα trace superposition of nsp14 N7-MTase_SAM_ (7TW7), nsp14 N7-MTase_SGC0946_ and nsp14 N7-MTase_SGC8158_.
(PDF)

S2 Fig Structural comparison of nsp14-N7-MTase. **(A)** Superposition of SARS-CoV-2 nsp14-N7-MTase_SGC0946_ (cyan) with SARS-CoV nsp14/nsp10_GpppA:SAH_ (PDB:5C8S, chain D, salmon). The GpppA carbon atoms are colored green and the SGC0946 carbon atoms are colored magenta. For clarity, the hinge region from SARS-CoV nsp14/nsp10_GpppA:SAH_ is not displayed. **(B)** Superposition of SARS-CoV-2 nsp14-N7-MTase_SGC0946_ (cyan) with SARS-CoV-2 nsp14 (PDB:7R2V, chain A, yellow). The hinge region is colored orange and the residues from it that may potentially interact with SGC0946 are shown in stick conformation. **(C)** Conformational flexibility of SGC0946 in SARS-CoV-2 nsp14-N7-MTase, DOT1L (PDB:4ER6) and CamA (7RFL) complex structures. **(D)** Superposition of SARS-CoV-2 nsp14-N7-MTase_SGC8158_ with SARS-CoV nsp14/nsp10_GpppA:SAH_ complex (PDB:5C8S, chain D). The GpppA carbon atoms are colored green and the SGC8158 carbon atoms are colored magenta. For clarity, the hinge region from SARS-CoV nsp14/nsp10_GpppA:SAH_ is not displayed. **(E)** Superposition of SARS-CoV-2 nsp14-N7-MTase_SGC8158_ (cyan) with SARS-CoV-2 nsp14 structure (PDB:7R2V, chain A, yellow). The hinge region is colored orange and the residues from it that may potentially interact with SGC8158 are shown in stick conformation. **(F)** Conformational flexibility of SGC8158 in SARS-CoV-2 nsp14-N7-MTase, PRMT7 (PDB:6OGN) and CamA (PDB:7RFN) complex structures.
(PDF)
