# Supplementary figures and images for: Structures of SARS-CoV-2 N7-methyltransferase with DOT1L and PRMT7 inhibitors provide a platform for new antivirals
Source: PLoS Pathog. 2023 Jul 31;19(7):e1011546. doi: 10.1371/journal.ppat.1011546 (PMC10414583; doi:10.1371/journal.ppat.1011546)

**A. nsp14-N7-MTase<sub>SGC0946</sub>**

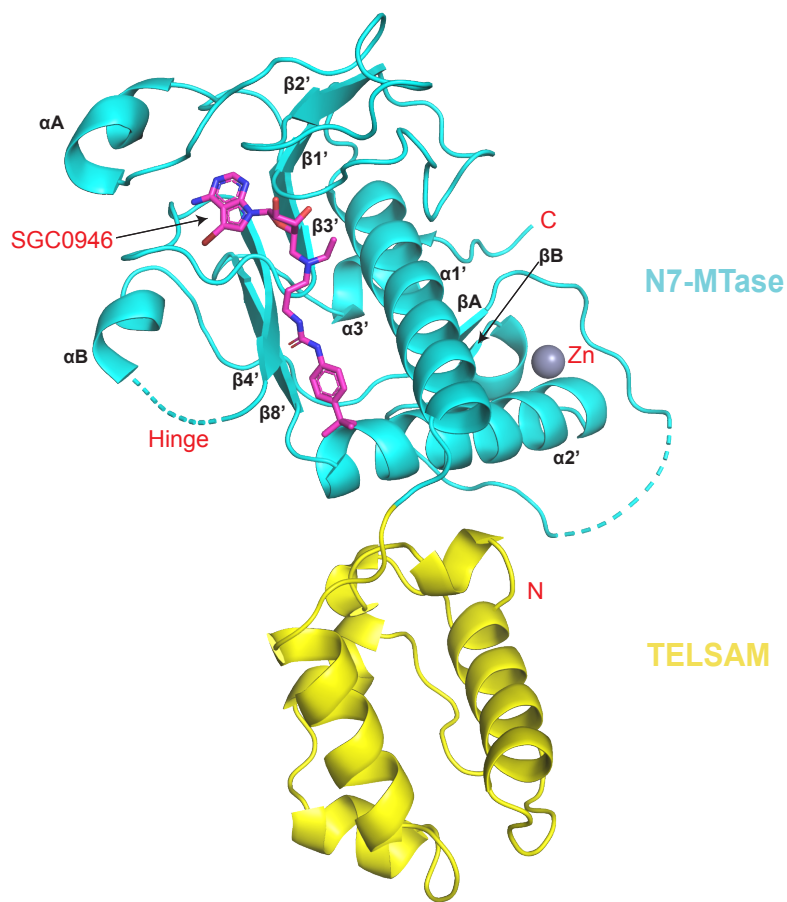

**B. nsp14-N7-MTase<sub>SGC8158</sub>**

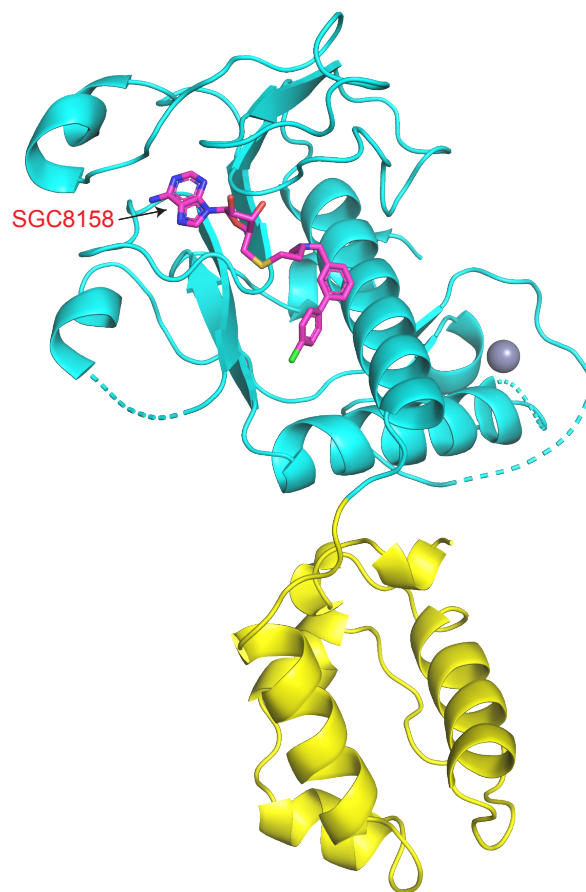

**C.**

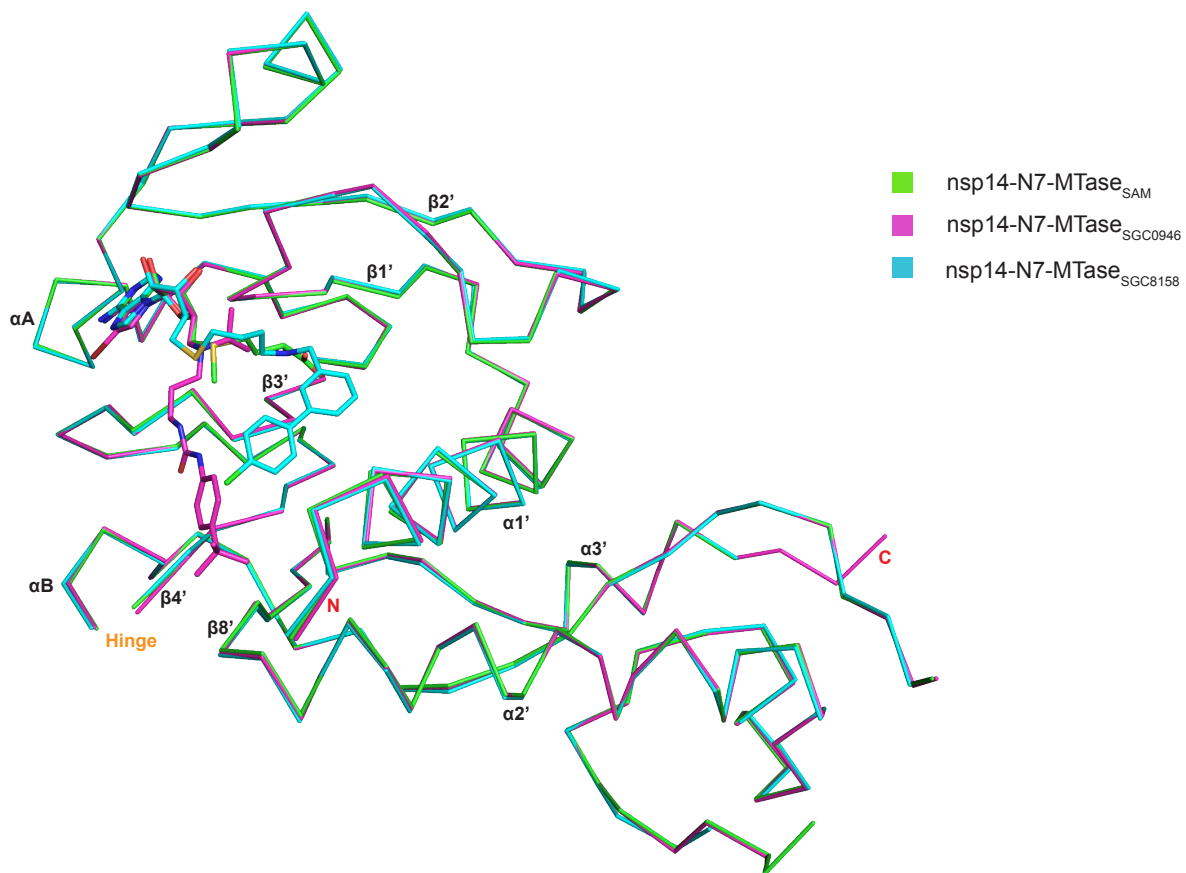

Supplement: S1 Fig — (A) Overall structure of SARS-CoV-2 nsp14-N7-MTaseSGC0946 complex fused with TELSAM. The nsp14 N7-MTase domain and TELSAM are colored in cyan and yellow, respectively. (B) Overall structure of SARS-CoV-2 nsp14-N7-MTaseSGC8158 complex fused with TELSAM. (C) Cα trace superposition of nsp14 N7-MTaseSAM (7TW7), nsp14 N7-MTaseSGC0946 and nsp14 N7-MTaseSGC8158. (PDF) [file ppat.1011546.s001.pdf]

**A.**

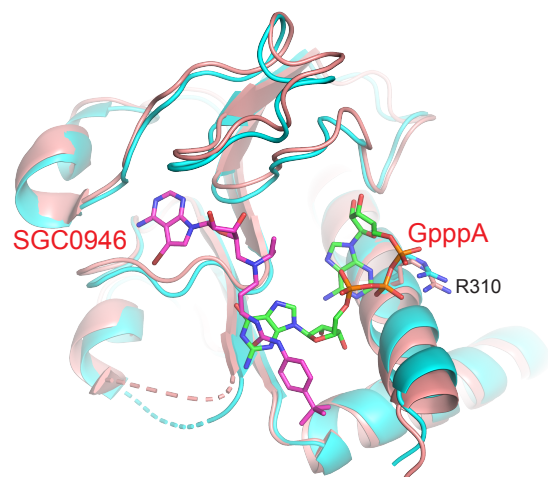

**D.**

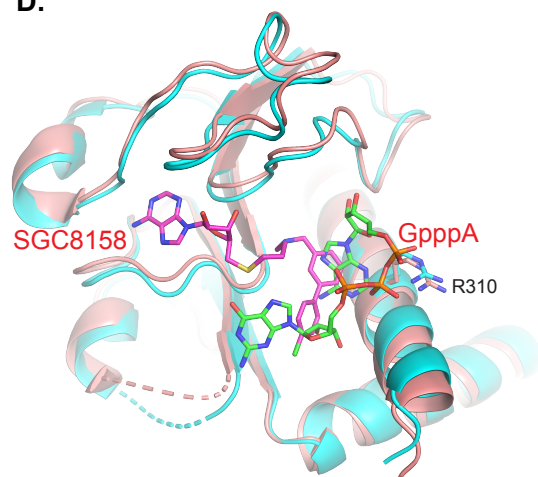

**B.**

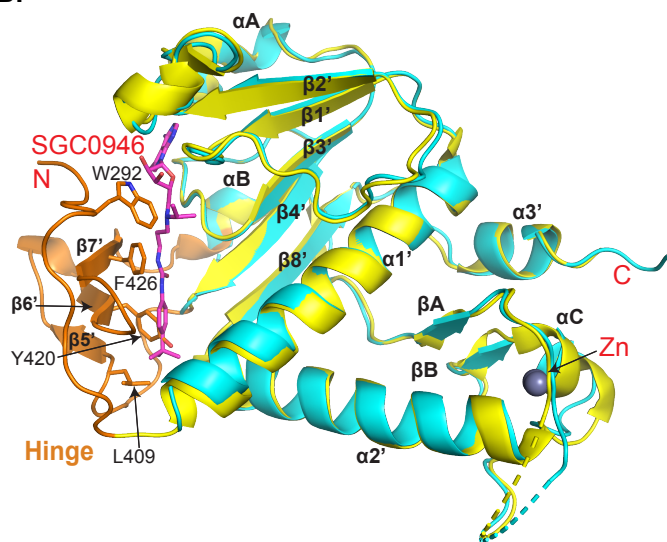

**E.**

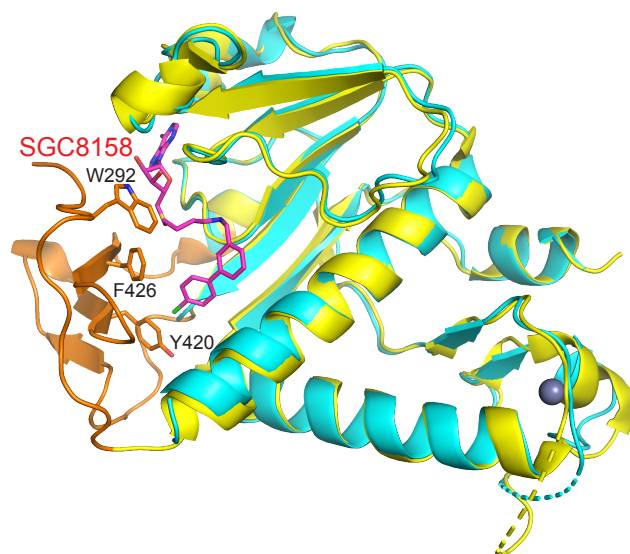

**C.**

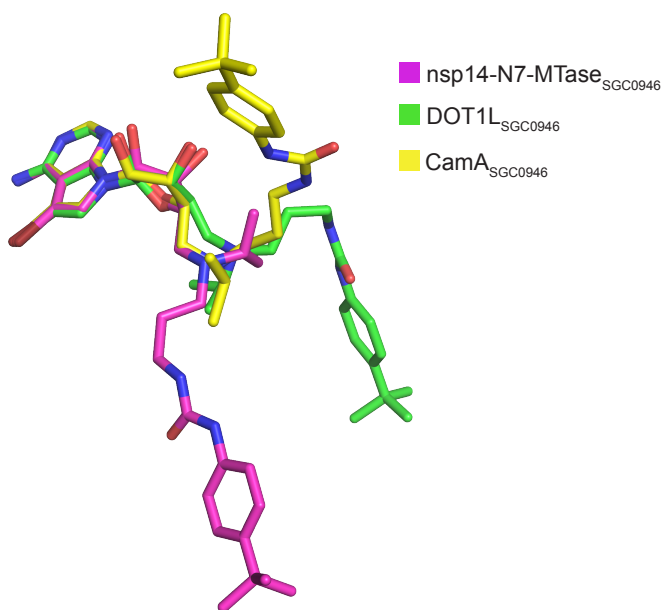

**F.**

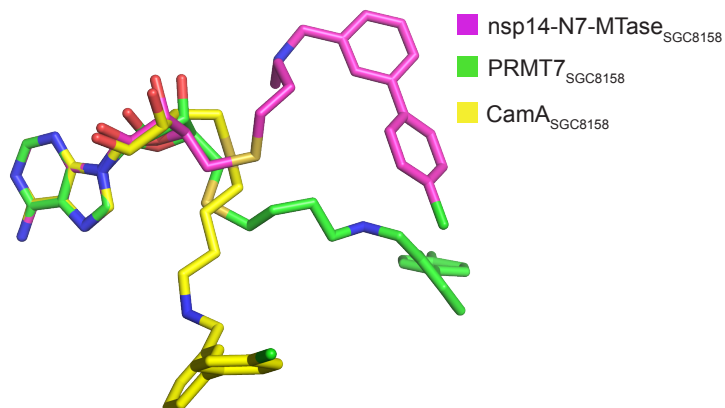

Supplement: S2 Fig — (A) Superposition of SARS-CoV-2 nsp14-N7-MTaseSGC0946 (cyan) with SARS-CoV nsp14/nsp10GpppA:SAH (PDB:5C8S, chain D, salmon). The GpppA carbon atoms are colored green and the SGC0946 carbon atoms are colored magenta. For clarity, the hinge region from SARS-CoV nsp14/nsp10GpppA:SAH is not displayed. (B) Superposition of SARS-CoV-2 nsp14-N7-MTaseSGC0946 (cyan) with SARS-CoV-2 nsp14 (PDB:7R2V, chain A, yellow). The hinge region is colored orange and the residues from it that may potentially interact with SGC0946 are shown in stick conformation. (C) Conformational flexibility of SGC0946 in SARS-CoV-2 nsp14-N7-MTase, DOT1L (PDB:4ER6) and CamA (7RFL) complex structures. (D) Superposition of SARS-CoV-2 nsp14-N7-MTaseSGC8158 with SARS-CoV nsp14/nsp10GpppA:SAH complex (PDB:5C8S, chain D). The GpppA carbon atoms are colored green and the SGC8158 carbon atoms are colored magenta. For clarity, the hinge region from SARS-CoV nsp14/nsp10GpppA:SAH is not displayed. (E) Superposition of SARS-CoV-2 nsp14-N7-MTaseSGC8158 (cyan) with SARS-CoV-2 nsp14 structure (PDB:7R2V, chain A, yellow). The hinge region is colored orange and the residues from it that may potentially interact with SGC8158 are shown in stick conformation. (F) Conformational flexibility of SGC8158 in SARS-CoV-2 nsp14-N7-MTase, PRMT7 (PDB:6OGN) and CamA (PDB:7RFN) complex structures. (PDF) [file ppat.1011546.s002.pdf]
